# Combined Metabolic Targeting With Metformin and the NSAIDs Diflunisal and Diclofenac Induces Apoptosis in Acute Myeloid Leukemia Cells

**DOI:** 10.3389/fphar.2018.01258

**Published:** 2018-11-02

**Authors:** Kathrin Renner, Anton Seilbeck, Nathalie Kauer, Ines Ugele, Peter J. Siska, Christina Brummer, Christina Bruss, Sonja-Maria Decking, Matthias Fante, Astrid Schmidt, Kathrin Hammon, Katrin Singer, Sebastian Klobuch, Simone Thomas, Eva Gottfried, Katrin Peter, Marina Kreutz

**Affiliations:** ^1^Department of Internal Medicine III, University Hospital Regensburg, Regensburg, Germany; ^2^Regensburg Center for Interventional Immunology (RCI), Regensburg, Germany

**Keywords:** metabolism, acute myeloid leukemia, AML, diclofenac, diflunisal, metformin, apoptosis

## Abstract

The accelerated metabolism of tumor cells, inevitable for maintaining high proliferation rates, is an emerging target for tumor therapy. Increased glucose and lipid metabolism as well as mitochondrial activity have been shown in solid tumors but also in leukemic cells. As tumor cells are able to escape the blockade of one metabolic pathway by a compensatory increase in other pathways, treatment strategies simultaneously targeting metabolism at different sites are currently developed. However, the number of clinically applicable anti-metabolic drugs is still limited. Here, we analyzed the impact of the anti-diabetic drug metformin alone or in combination with two non-steroidal anti-inflammatory drugs (NSAIDs) diclofenac and diflunisal on acute myeloid leukemia (AML) cell lines and primary patient blasts. Diclofenac but not diflunisal reduced lactate secretion in different AML cell lines (THP-1, U937, and KG-1) and both drugs increased respiration at low concentrations. Despite these metabolic effects, both NSAIDs showed a limited effect on tumor cell proliferation and viability up to a concentration of 0.2 mM. In higher concentrations of 0.4–0.8 mM diflunisal alone exerted a clear effect on proliferation of AML cell lines and blocked respiration. Single treatment with the anti-diabetic drug metformin blocked mitochondrial respiration, but proliferation and viability were not affected. However, combining all three drugs exerted a strong cytostatic and cytotoxic effect on THP-1 cells. Comparable to the results obtained with THP-1 cells, the combination of all three drugs significantly reduced proliferation of primary leukemic blasts and induced apoptosis. Furthermore, NSAIDs supported the effect of low dose chemotherapy with cytarabine and reduced proliferation of primary AML blasts. Taken together we show that low concentrations of metformin and the two NSAIDs diclofenac and diflunisal exert a synergistic inhibitory effect on AML proliferation and induce apoptosis most likely by blocking tumor cell metabolism. Our results underline the feasibility of applying anti-metabolic drugs for AML therapy.

## Introduction

Acute myeloid leukemia (AML) is a heterogeneous group of neoplastic disorders characterized by the accumulation of myeloid blasts in the bone marrow and blood as well as an arrest in differentiation. AML represents the most common acute leukemia in adults and is still a mostly incurable and lethal disease in the majority of patients. Induction therapy with cytarabine and anthracyclines is highly effective in killing leukemic cells and has largely remained unchanged since the 1970s (Perl, 2018). Despite a high rate of complete remissions, the overall survival is very poor as many patients relapse. Relapses are caused by regrowth of chemotherapy-resistant leukemic cells.

New studies expanded the knowledge on AML heterogeneity and identified new molecular groups. These advances are now translated into several new targeted therapies (Perl, 2017). Mutations in isocitrate dehydrogenase (IDH)1 or IDH2 are detected in approximately 15% of AML patients. Both enzymes are important for cell energy metabolism and catalyze the interconversion of isocitrate into α-ketoglutarate. AML therapy with oral, small-molecule inhibitors of mutant IDH1 and mutant IDH2, respectively, has shown promising results (Stein et al., 2017; DiNardo et al., 2018) indicating that metabolic targeting is an effective approach for AML therapy.

In solid tumors it is well-established that oncogenes reprogram tumor metabolism and accelerate glucose metabolism. This phenomenon, known as the “Warburg effect,” is associated with an aggressive phenotype and poor prognosis in many tumor entities (Renner et al., 2017). Ju et al. (2017) described that internal tandem duplication (ITD) mutation in fms like tyrosine kinase (FLT) 3 (FLT3/ITD), which is detected in about 30% of AML patients, promotes the Warburg effect. Leukemia cell lines overexpressing FLT3/ITD exhibited increased glucose uptake and lactate secretion. In line, the commonly used glycolytic inhibitor 2-deoxyglucose (2-DG) potentiated the anti-leukemia effect of sorafenib. These data suggest that combination of metabolic intervention and conventional chemotherapy could be exploited as therapeutic strategy in patients with FLT3/ITD mutation (Ju et al., 2017).

In line, inhibition of glycolytic metabolism by 2-DG increased the efficacy of Aurora kinase inhibitors in AML cell lines (Liu et al., 2013) and potentiated the cytotoxicity of arabinofuranosyl cytidine (Chen et al., 2014).

Many hypotheses tried to explain the therapeutic resistance in AML patients. A linkage between reduced sensitivity to chemotherapy and accelerated glycolysis has been described in a murine AML model. Here, chemotherapy-resistant AML exhibited increased lactate production and fewer mitochondria (Nobrega-Pereira et al., 2018). In contrast, another study demonstrated that cytarabine-resistant cells displayed a shift to fatty acid oxidation and increased oxidative phosphorylation (OXPHOS) (Farge et al., 2017). Similar results were obtained by Qian et al. (2016) showing that high expression of TP53-induced glycolysis and apoptosis regulator (TIGAR), which inhibits glycolysis, was associated with poor survival and high incidence of relapse. Furthermore, Gallipoli et al. (2018) showed that FLT3/ITD AML depends on glutaminolysis which supports mitochondrial activity.

Vascular endothelial growth factor (VEGF)-signaling seems to be associated with changes in metabolism and chemoresistance. Blocking VEGF receptor 2 (VEGFR2) induced mitochondrial biogenesis and increased the vulnerability of leukemic cells (Nobrega-Pereira et al., 2018). VEGF-C induces cyclooxygenase (COX)-2 expression in AML cell lines and COX-2 inhibition limits tumor cell proliferation *in vitro* and suppresses xenograft tumor formation (Zhang et al., 2013; Hua et al., 2014). Moreover, combination of celecoxib, a COX-2 inhibitor, with doxorubicin revealed a synergistic effect on growth inhibition and apoptosis induction in the AML cell line HL-60 and primary AML cells (Chen et al., 2013).

These results suggest that AML cells are heterogeneous in their metabolic profile and use different metabolic pathways to fuel proliferation and acquire resistance to chemotherapy. Here we investigated the impact of three inhibitors which target glycolysis, COX, and OXPHOS alone and in combination on AML cell lines and primary human AML blasts. Our study illustrates that simultaneous targeting of different metabolic pathways may represents a powerful therapeutic strategy for AML patients.

## Materials and Methods

### Chemicals and Drugs

All drugs were purchased from Sigma-Aldrich (St Louis, MO, United States) and dissolved in water, unless otherwise indicated. The sodium salt of diclofenac (Fagron, Barsbüttel, Germany) and metformin hydrochloride (Sigma-Aldrich) were dissolved in culture medium. Diflunisal (Fluka, Munich, Germany) was dissolved in 20 mM arginine containing water and cytarabine (Stada, Bad Vilbel, Germany) in 0.11 mM sodium lactate solution.

### Cells and Cell Culture

The cell lines U937 (human histiocytic leukemia, DSMZ) and THP-1 (monocytic leukemia cell line, DSMZ) were cultured in RPMI 1640, 10% fetal calf serum (both from PAN Biotech, Aidenbach, Germany), 2 mM glutamine, 50 U/mL penicillin/50 μg/mL streptomycin (all from Gibco/Life Technologies, Carlsbad, CA, United States) at 5% CO2 and 37°C.

Primary AML blasts were obtained from patients after written informed consent. The study was approved by the Institutional Ethics Committee of the University Hospital of Regensburg and designed and conducted in accordance with the Declaration of Helsinki (ethic vote 05-097). AML blasts were cultured in RPMI 1640, 10% fetal calf serum (both from PAN), 10% human AB serum, 2 mM glutamine, 50 U/mL penicillin/50 μg/mL streptomycin (all from Gibco), 20 ng/mL IL-3, G-CSF as well as TPO (all from Peprotech, Hamburg, Germany). 5 × 10^5^ cells/mL medium were seeded in 24-well plates at 5% CO2 and 37°C. Every 3–4 days, medium was changed.

### Determination of Cell Proliferation

To measure proliferation of cell lines, 3 × 10^4^ cells/0.2 mL medium were seeded into flat-bottom 96-well plates with indicated concentrations of diclofenac, diflunisal, metformin and cytarabine. To analyze immediate anti-proliferative effects, 0.5 μCi/0.2 mL ^3^H-thymidine (Amersham Pharmacia, Piscataway, NJ, United States) was added after 2 h and ^3^H-thymidine incorporation was determined after 24 h. In a second set of experiments, cells were labeled after 24 h and cultured for another after 24 h (48 h total).

### Determination of Apoptosis

For analysis of apoptosis, 3–5 × 10^5^ cells/mL medium were treated with diclofenac, diflunisal, metformin and cytarabine for 24 h or 48 h. After treatment, cells were stained with Annexin-V-FITC and 7-aminoactinomycin D (7-AAD) (both from BD Biosciences, Franklin Lakes, NJ, United States) according to the manufacturer’s instructions. Flow cytometric analyses were performed on a FACSCalibur (BD Biosciences) using BD CellQuestPro for data acquisition and analysis. Final processing and analysis was performed with FlowJo v9.5.3 software (FlowJo, LLC, Ashland, OR, United States).

### Determination of Lactate in Tumor Cell Supernatants

Cells were seeded into 24-well plates at a concentration of 5 × 10^5^ cells/ mL medium with or without diclofenac, diflunisal, metformin and cytarabine. After 24 or 48 h, lactate levels in cell culture supernatants were determined with a Dimension Vista (Siemens, Munich, Germany) using reagents from Roche (Mannheim, Germany) at the Department of Clinical Chemistry, University Clinic, Regensburg, Germany.

### Online-Measurement of Oxygen Concentration in Cell Culture

The SDR SensorDish^®^ Reader (PreSens Precision Sensing GmbH, Regensburg, Germany) is a 24-channel oxygen and pH meter. The optical oxygen (OxoDish^®^) sensor is integrated at the bottom of each well of a 24-well multidish. The sensors are luminescent dyes embedded in an analyte-sensitive polymer. The luminescence lifetime of these dyes depends on the amount of analyte. The sensors are read out non-invasively through the bottom of the multidish by the SensorDish^®^ Reader. The resulting signal is converted automatically to the respective parameter (dissolved oxygen) using calibration parameters stored in the software. 5 × 10^5^ cells/mL medium were incubated with or without diclofenac, diflunisal, metformin and cytarabine. The SensorDish^®^ Reader was used in the incubator for the whole duration of the 16-h cultivation period and measurements were performed in 30–60 s intervals.

### Statistical Analysis

All results represent mean ± standard error (SEM) of at least three independent experiments. Significance was determined by ANOVA and *post hoc* by Holm-Sidak’s multiple comparisons test, ^∗∗∗^*p* < 0.001; ^∗∗^*p* < 0.01; and ^∗^*p* < 0.05.

## Results

Tumor metabolism supports tumor growth and is associated with chemotherapy resistance. Targeting tumor metabolism is an attractive option to support conventional tumor therapy. Here we investigated the impact of anti-metabolic drugs alone or in combination on human AML cell lines and primary AML blasts.

### Impact of Metformin, NSAIDs and 2-DG on Metabolism and Proliferation of AML Cell Lines

**FIGURE 1:**
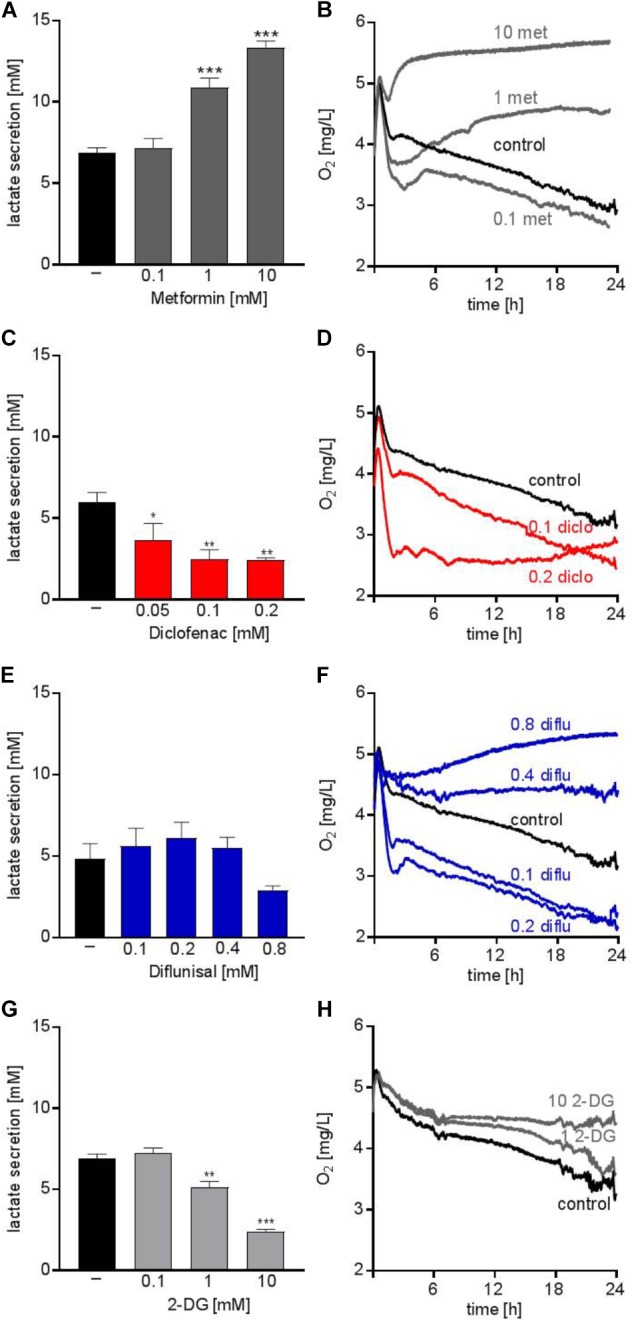
Metabolic effects of single treatment with metformin, diclofenac, diflunisal and 2-DG on THP-1 cells. **(A,C,E,G)** Cells were seeded in 24-well plates at a concentration of 5 × 10^5^ cells/ mL medium with or without metformin, diclofenac, diflunisal and 2-DG in the indicated concentrations. After 24 h, cumulative lactate levels in cell culture supernatants were determined. **(B,D,F,H)** Cells were seeded in the same density in 24-well dishes with integrated optical oxygen (OxoDish^®^) sensors in the bottom of each well. The SensorDish^®^ Reader was placed in the incubator for 24 h and measurements were performed in 30–60 s intervals. **(A,C,E,G)** data are shown as mean ± SEM (*n* ≥ 3), ^∗^*P* < 0.05, ^∗∗^*P* < 0.01, and ^∗∗∗^*P* < 0.001 significance was calculated by ANOVA and *post hoc* Holm-Sidak’s multiple comparisons test. **(B,D,F,H)** data are shown as mean (*n* ≥ 3 with the exception of **(F)** 0.4–0.8 mM diflunisal *n* = 2).

The anti-diabetic drug metformin lowers OXPHOS via inhibiting complex I of the electron transport chain in the mitochondria. Several studies addressed the anti-cancer potential of metformin *in vitro* and in clinical trials, however, mainly in solid tumors (Pollak, 2014). Metformin accelerated glucose metabolism and increased lactate secretion in the human AML cell line THP-1 starting from concentrations of 1 mM (Figure 1A). In parallel, oxygen consumption was blocked (Figure 1B). Diclofenac, a non-steroidal anti-inflammatory drug (NSAID) which has been shown to inhibit glucose metabolism (Gottfried et al., 2013) significantly decreased lactate secretion starting from 0.05 mM and increased oxygen consumption (Figures 1C,D). Another NSAID, diflunisal, exhibited no effect on lactate secretion but increased oxygen consumption in concentrations of 0.1–0.2 mM (Figures 1E,F). However, at higher diflunisal concentrations of 0.4–0.8 mM, OXPHOS was inhibited (Figure 1F). In comparison, we tested a classical glycolytic inhibitor, 2-deoxyglucose (2-DG). A decrease in lactate secretion was obtained with high 2-DG concentrations of 1–10 mM (Figure 1G); OXPHOS inhibition occurred at 10 mM 2-DG (Figure 1H).

**FIGURE 2:**
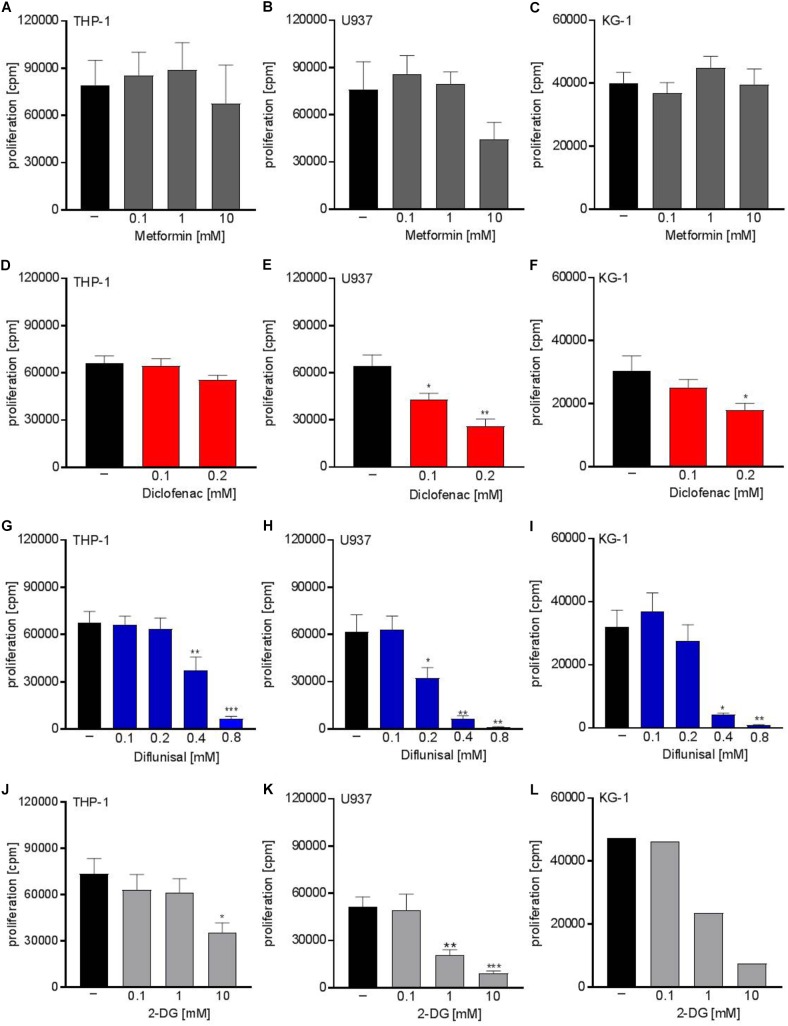
Cytostatic effects of single treatment with metformin, diclofenac, diflunisal and 2-DG on the proliferation of myeloid leukemic cell lines. To analyze effects on proliferation of **(A,D,G,J)** THP-1, **(B,E,H,K)** U937 and **(C,F,I,L)** KG-1, 3 × 10^4^ cells/0.2 mL medium were seeded in 96-well plates with indicated concentrations of metformin, diclofenac, diflunisal and 2-DG. ^3^H-thymidine was added after 2 h and ^3^H-thymidine incorporation was determined after 20–22 h. **(A–K)** data are shown as mean ± SEM (*n* ≥ 4), ^∗^*P* < 0.05, ^∗∗^*P* < 0.01, and ^∗∗∗^*P* < 0.001 significance was calculated by ANOVA and *post hoc* Holm-Sidak’s multiple comparisons test. **L** data are shown as mean (*n* = 2).

Next we analyzed whether metabolic targeting modulates cell growth of different AML cell lines. Even though metformin significantly blocked respiration at doses of 1-10 mM, no significant effect on proliferation was detected for THP-1, U937, and KG-1 AML cell lines (Figures 2A–C). This could in part be because decreased OXPHOS was compensated by increased glycolytic activity as shown by increased lactate secretion after metformin treatment (Figure 1A). Proliferation was not significantly lowered by diclofenac in THP-1 cells (Figure 2D) whereas U937 and KG-1 were more susceptible to diclofenac treatment resulting in decreased proliferation (Figures 2E,F). Diflunisal significantly inhibited proliferation in all three AML cell lines (Figures 2G–I) starting from a concentration of 0.4 mM where OXPHOS was clearly blocked. In contrast to metformin, AML cells treated with diflunisal showed no compensation regarding glycolytic activity as lactate production was not altered after diflunisal treatment. (Figure 1E). 2-DG also inhibited proliferation at concentrations of 10 mM where both OXPHOS and glycolysis was blocked (Figures 2J–L). These data indicate that metabolic targeting only results in robust proliferation arrest when glycolysis and OXPHOS are targeted simultaneously.

### Combined Metabolic Targeting Limits Proliferation of Leukemia Cell Lines and Primary Blasts

**FIGURE 3:**
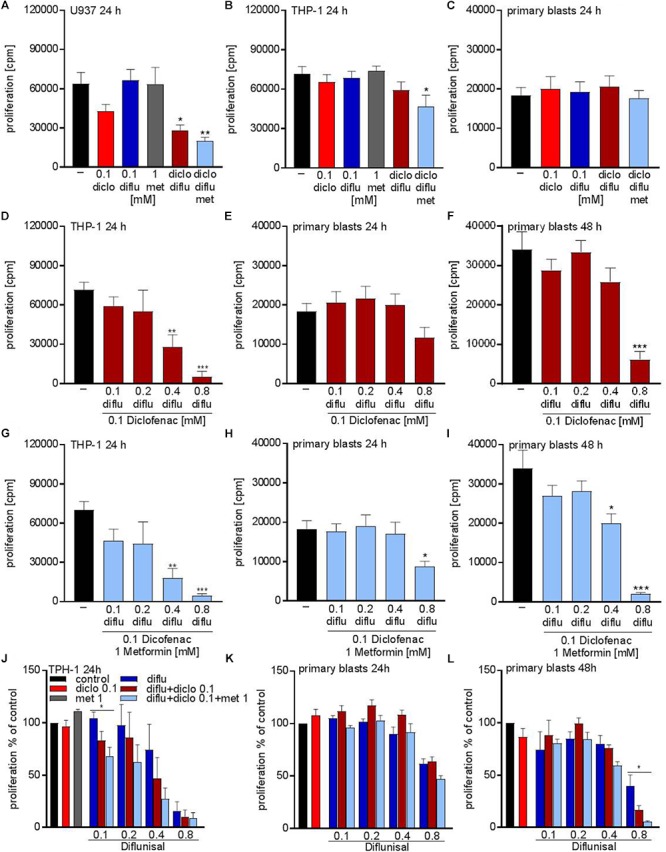
Cytostatic effects after combined treatment with metformin, diclofenac and diflunisal on AML cells. 3 × 10^4^cells/0.2 mL medium **(A)** U937 cells or **(B)** THP-1 cells or **(C)** primary blasts were seeded in 96-well plates with 1 mM metformin, 0.1 mM diclofenac and 0.1 mM diflunisal alone or in the indicated combinations. ^3^H-thymidine incorporation was determined after 24 h. In a second set of experiments we increased the diflunisal concentration. **(D)** THP-1 cells or **(E)** primary blasts were incubated with 0.1 mM up to 0.8 mM diflunisal plus low dose 0.1 mM diclofenac for 24 h. **(F)** alternatively, the incubation period of primary blasts was prolonged to 48 h. **(G–I)** in another set of experiments the triple combination with diclofenac and 1 mM metformin was used. Proliferation under single drug treatment or the various drug combinations was normalized to respective controls and is shown for **(J)** THP-1 cells and primary blasts treated for **(K)** 24 h or **(L)** 48 h. data are shown as mean ± SEM (*n* ≥ 3), ^∗^*P* < 0.05, ^∗∗^*P* < 0.01, and ^∗∗∗^*P* < 0.001 significance was calculated by ANOVA and *post hoc* Holm-Sidak’s multiple comparisons test.

Next we tried to combine low physiological doses of the drugs to augment the effect compared to the single treatment. Combination of 0.1 mM of diclofenac and 0.1 mM diflunisal, clearly suppressed the proliferation of U937 but not in THP-1 and primary AML blasts (Figures 3A–C). Addition of 1 mM metformin, which as a single drug had no impact on proliferation, could overcome the resistance of THP-1 against NSAIDs and the combined treatment significantly lowered the proliferation rate of THP-1 cells but not primary AML blasts (Figures 3B,C).

As the salicylic acid derivative diflunisal can be administered to patients in 10-fold higher concentrations compared to diclofenac, we increased the diflunisal dose and combined it with low concentrations of diclofenac and metformin (Figures 3D–I). As shown in Figure 2G, diflunisal alone inhibited THP-1 proliferation at a concentration of 0.4–0.8 mM. Combined treatment with diclofenac and metformin could not further decrease the proliferation rate of THP-1 cells (Figures 3D,G). In primary AML blasts no significant effect was detected with diflunisal plus diclofenac (Figure 3E) after 24 h, but addition of metformin reduced proliferation (Figure 3H). When we prolonged the treatment to 48 h, this effect was even more pronounced and also 0.4 mM diflunisal as triple combination therapy significantly reduced proliferation of primary blasts (Figure 3I). For direct comparison we normalized the proliferation under single drug treatment and the various combinations to untreated cells (Figures 3J–L). Taken together, these data prove that combination of two or three treatments which alone were not effective resulted in cytostatic effects not only in cell lines but also primary blasts.

### Metabolic Effects of NSAIDs and Metformin on Primary AML Blasts

**FIGURE 4:**
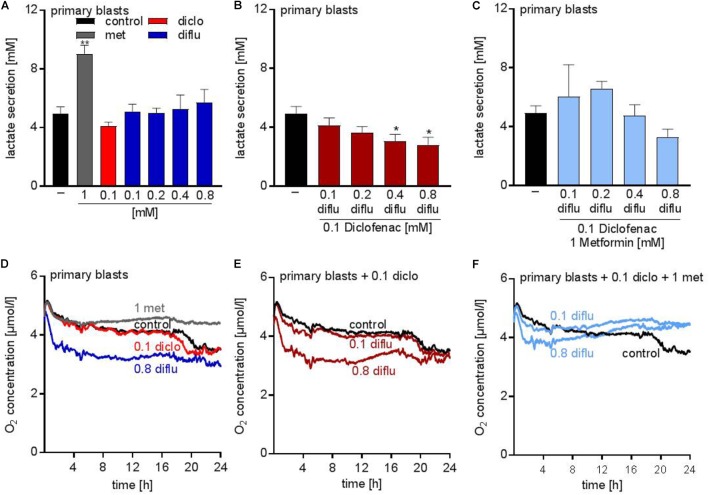
Metabolic effects of treatment with metformin, diclofenac and diflunisal on primary blasts. AML blasts were seeded in 24-well plates at a concentration of 5 × 10^5^ cells/mL medium with or without metformin, diclofenac and diflunisal and 2-DG in the indicated concentrations. **(A–C)** After 24 h, cumulative lactate levels in cell culture supernatants were determined. **(D–F)** Cells were seeded in the same density in 24-well dishes with integrated optical oxygen (OxoDish^®^) sensors in the bottom of each well. The SensorDish^®^ Reader was placed in the incubator for 24 h and measurements were performed in 30–60 s intervals. **A,D** show single treatments; in **B,E** diflunisal was combined with 0.1 mM diclofenac and in **C,F** triple treatments of diflunisal, diclofenac and metformin are shown. **(A–C)** data are shown as mean ± SEM (*n* ≥ 3), ^∗^*P* < 0.05 and ^∗∗^*P* < 0.01; significance was calculated by ANOVA and *post hoc* Holm-Sidak’s multiple comparisons test. **(D–F)** data are shown as mean (*n* = 3).

Analysis of the metabolic effects of the drugs on AML blasts revealed similar results compared to the leukemia cell lines. Metformin accelerated lactate secretion whereas diclofenac alone or in combination with diflunisal reduced lactate secretion (Figures 4A,B). Combination of all three drugs resulted in a slightly reduced lactate secretion even though metformin alone doubled the lactate secretion (Figures 4A,C). Furthermore, oxygen consumption was determined with the Presens technology. In contrast to THP-1 cells (Figure 1F), diflunisal increased oxygen consumption in primary blasts even in high diflunisal concentrations of 0.8 mM (Figure 4D). Diclofenac alone or in combination with 0.1 mM diflunisal had no effect on oxygen consumption (Figures 4D,E). Metformin again inhibited respiration alone and in combination with the NSAIDs (Figures 4D,F) thereby counteracting the effect of diflunisal on OXPHOS.

### Apoptosis Induction by Combined Metabolic Targeting

**FIGURE 5:**
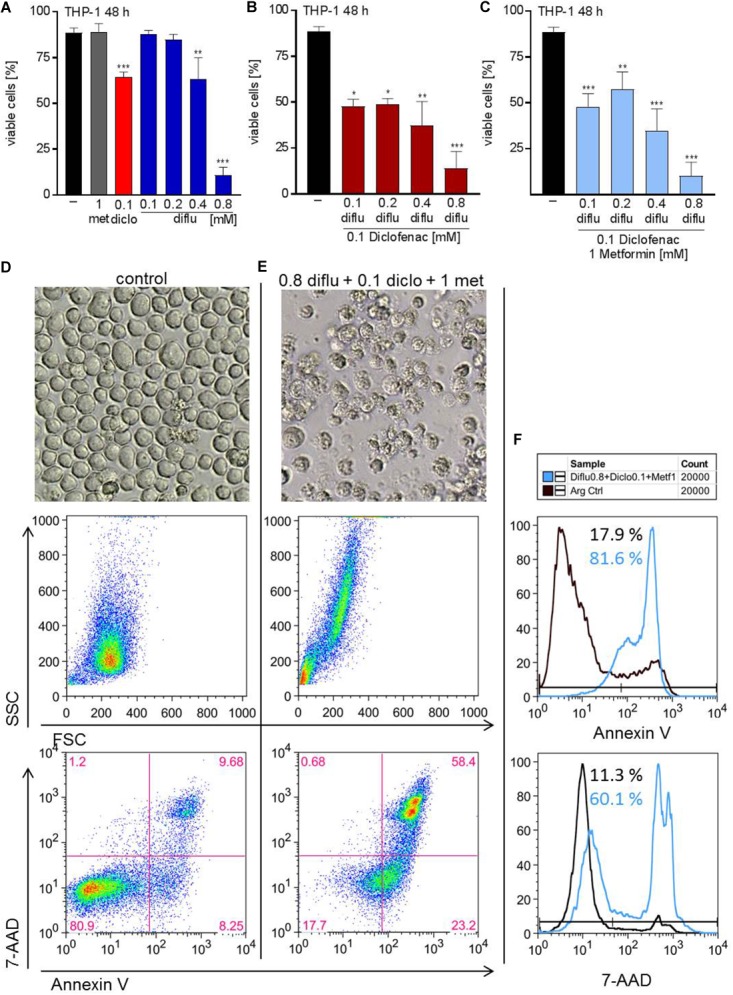
Impact of metformin, diclofenac and diflunisal on apoptosis in THP-1 cells. For analysis of apoptosis, 3–5 × 10^5^ cells/mL medium were treated with **(A)** single drugs or **(B)** combination of increasing concentrations of diflunisal (0.1–0.8 mM) plus 0.1 mM diclofenac or **(C,E)** triple combinations for 48 h. After treatment, pictures were taken to document the morphology of **(D)** untreated or **(E)** treated cells; one representative experiment is shown. Cells were stained for Annexin-V-FITC and 7-aminoactinomycin D (7-AAD) and analyzed by flow cytometry. Percentage of Annexin-V single positive cells (lower right quadrant), regarded as early apoptotic cells, 7-AAD single positive cells (upper left quadrant), regarded as necrotic cells or Annexin V/7-AAD double positive cells (upper right quadrant), representing late apoptotic cells, can be depicted either from scatter blots or histograms **(D,E)** Representative scatter blots and **(F)** histograms are presented. **(A–C)** data are shown as mean ± SEM (*n* = 3), ^∗^*P* < 0.05, ^∗∗^*P* < 0.01, and ^∗∗∗^*P* < 0.001; significance was calculated by ANOVA and *post hoc* Holm-Sidak’s multiple comparisons test.

To distinguish proliferation arrest from the induction of cell death, we incubated THP-1 cells with the drugs alone and in combination and stained with annexin-V/7-AAD to determine apoptosis. Low concentrations of diclofenac but not diflunisal and metformin induced apoptosis, however, high diflunisal concentration of 0.8 mM had a strong effect on cell viability (Figure 5A). The combination of low concentrations of diclofenac and diflunisal induced apoptosis (Figure 5B). Metformin had no effect and could not further support the effect of diclofenac and diflunisal (Figure 5C). Morphology and original flow cytometry data presented either as scatter blots or histograms of Annexin-V/7-AAD staining of THP-1 cells under treatment are shown in Figures 5D–F. Percentage of Annexin-V single positive cells (lower right quadrant), regarded as early apoptotic cells, 7-AAD single positive cells (upper left quadrant), regarded as necrotic cells or Annexin V/7-AAD double positive cells (upper right quadrant), representing late apoptotic cells, can be depicted either from scatter blots or histograms (Figures 5D–F).

**FIGURE 6:**
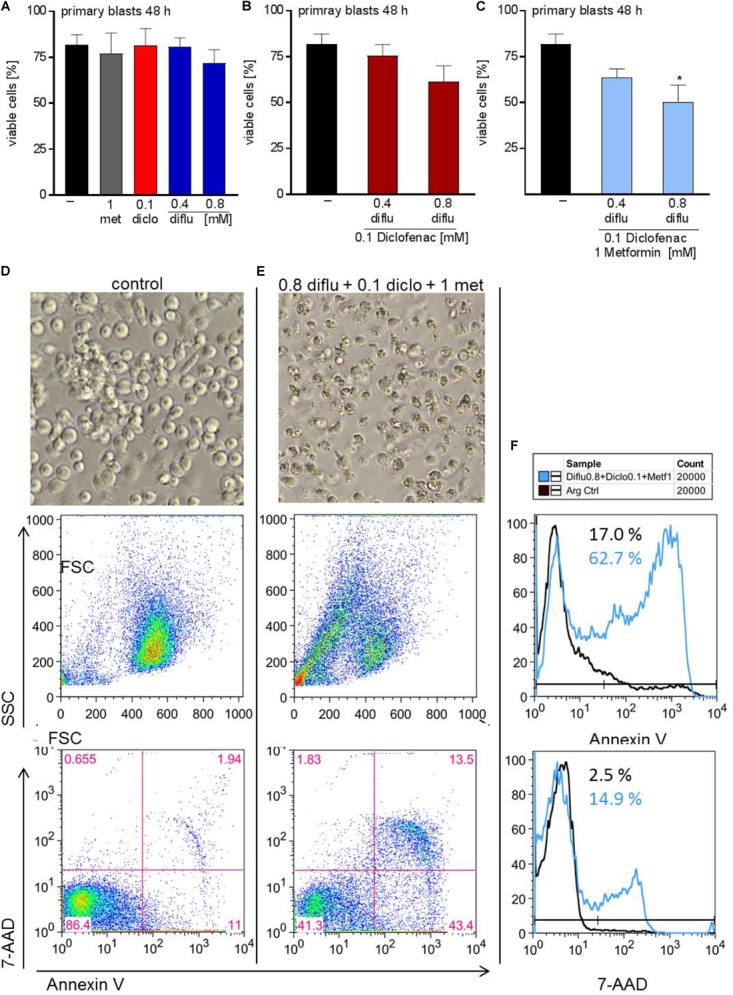
Impact of metformin, diclofenac and diflunisal on apoptosis in primary AML blasts. For analysis of apoptosis, 3–5 × 10^5^ cells/mL medium were treated with **(A)** single drugs or **(B)** combination of diflunisal (0.4 mM, 0.8 mM) plus 0.1 mM diclofenac or **(C,E)** triple combinations for 48 h. After treatment, pictures were taken to document the morphology of **(D)** untreated or **(E)** treated cells; one representative experiment is shown. Then AML blasts were stained with Annexin-V-FITC and 7-aminoactinomycin D (7-AAD) and analyzed by flow cytometry. Percentage of Annexin-V single positive cells (lower right quadrant), regarded as early apoptotic cells, 7-AAD single positive cells (upper left quadrant), regarded as necrotic cells or Annexin V/7-AAD double positive cells (upper right quadrant), representing late apoptotic cells, can be depicted either from scatter blots or histograms **(D,E)** Representative scatter blots and **(F)** histograms are presented. **(A–C)** data are shown as mean ± SEM (*n* = 3), ^∗^*P* < 0.05; significance was calculated by ANOVA and *post hoc* Holm-Sidak’s multiple comparisons test.

In contrast to THP-1 cells, AML blasts did not respond to single drug treatment (Figure 6A). Combination of low doses of diclofenac with high concentrations of 0.8 mM diflunisal also had no effect (Figure 6B). Applying 0.8 mM diflunisal as triple therapy together with diclofenac and metformin resulted in apoptosis induction (Figures 6C–E). This effect is also clearly visible from the pictures, FCS/SSC characteristics and original data on Annexin-V/7-AAD staining, presented either as scatter blots or as histograms (Figures 6D–F).

### Metabolic Targeting in Combination With Metronomic Chemotherapy

**FIGURE 7:**
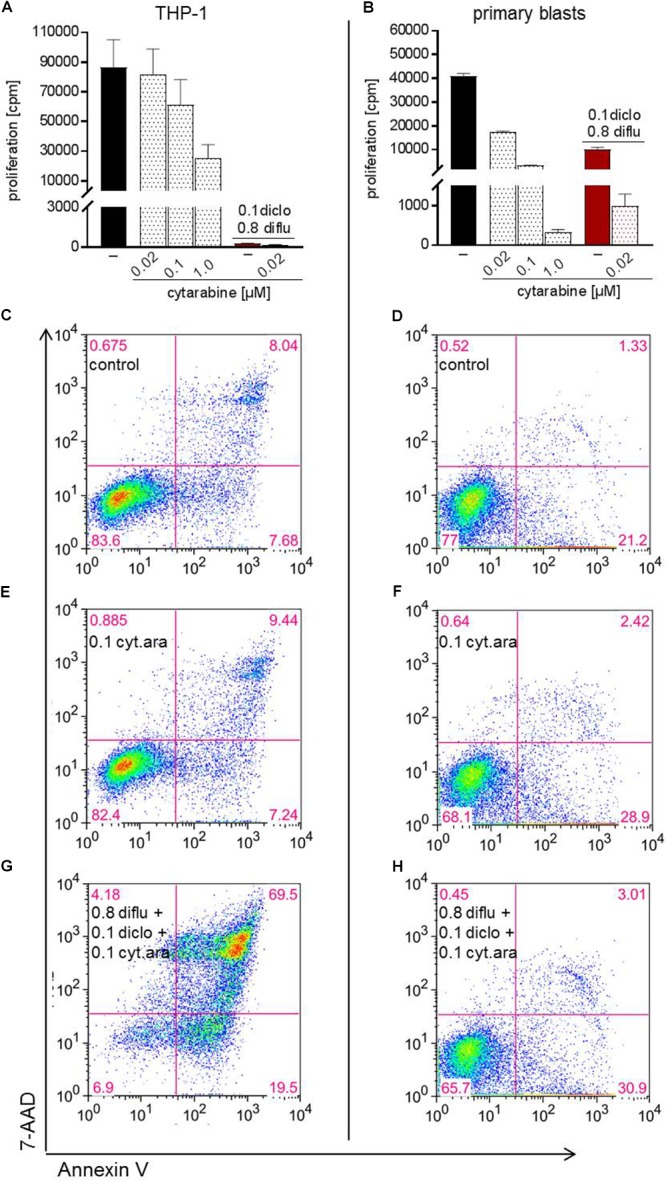
Impact of cytarabine in combination with diclofenac and diflunisal on proliferation and apoptosis in AML cells. To analyze effects on proliferation of **(A)** THP-1 or **(B)** primary blasts 3 × 10^4^ cells/0.2 mL medium were seeded in 96-well plates with indicated concentrations of cytarabine, diclofenac and diflunisal. ^3^H-thymidine was added after 24 h and ^3^H-thymidine incorporation determined after another 24 h. For analysis of apoptosis, 3–5 × 10^5^ cells/mL medium were treated with or without cytarabine as a (**C–F**) single drug or **(G,H)** triple combination of cytarabine with diflunisal and diclofenac for 48 h. Cells were stained with Annexin-V-FITC and 7-aminoactinomycin D (7-AAD) and analyzed by flow cytometry. **(A,B)** Data are shown as mean ± SEM (two independent experiments in triplicate for THP-1, one experiment in triplicate for AML blasts). **(C–H)** Apoptosis was analyzed in one experiment with either THP-1 or primary blasts.

Finally, we analyzed the impact of low dose standard chemotherapy with cytarabine alone and in combination with anti-metabolic treatment. In THP-1 cells and in primary blasts, cytarabine dose-dependently inhibited proliferation and the effect of cytarabine was more pronounced in primary blasts (Figures 7A,B). Triple combination of diclofenac, diflunisal and low dose cytarabine almost completely shut down proliferation of primary blasts and THP-1 cells (Figures 7A,B). However, low dose 0.02 and 0.1 μM cytarabine had little impact on apoptosis induction compared to untreated cells (data not shown and Figures 7C–F). Combination of low dose 0.1 μM cytarabine with diclofenac and diflunisal could induce apoptosis in THP-1 cells and a limited effect was observed for primary blasts (Figures 7G,H).

## Discussion

Cellular proliferation requires nutrient uptake, synthesis of building blocks and energy. Oncogenes that drive tumor cell proliferation directly or indirectly lead to metabolic changes; c-MYC and other oncogenes upregulate metabolite transporters and enzymes required for glucose metabolism (Vander Heiden and DeBerardinis, 2017). In line, the majority of tumor cells are characterized by an accelerated glucose metabolism and metabolize pyruvate into lactate even in the presence of adequate oxygen, a phenomenon known as “Warburg effect” (Warburg et al., 1927). Glycolysis generates building blocks to guarantee rapid tumor growth whereas OXPHOS provides energy.

Many classical anticancer agents inhibit nucleotide metabolism, but some chemotherapeutic drugs have also been shown to target tumor energy metabolism. Cyclophosphamide caused a drop in the glycolytic rate in a murine fibrosarcoma model (Poptani et al., 2003). Sorafenib, a multikinase inhibitor, has been shown to limit mitochondrial activity and resistance was associated with glycolytic reprogramming (Tesori et al., 2015). Therefore, metabolic changes occur in tumors during chemotherapy and seem to relate to the development of resistance.

Here, we analyzed the efficacy of drugs targeting glycolysis, COX, and OXPHOS alone and in combination on AML cell lines and primary human AML blasts.

Our data show that the glycolytic inhibitor 2-DG and low concentrations of the NSAID diclofenac both limit glycolysis and decrease lactate secretion of AML cell lines. Furthermore, both drugs modulated respiration; in low concentration diclofenac and diflunisal increased respiration. However, higher concentrations of diflunisal and 2-DG decreased oxygen consumption. In line, several other NSAIDs impair mitochondrial activity indicating that OXPHOS inhibition is a COX-related effect (Moreno-Sanchez et al., 1999). But the inhibition of lactate secretion by diclofenac seems not to be related to COX inhibition since diflunisal, another NSAID, had no effect on lactate production. In line, it has been shown that even high concentrations of aspirin have no effect on lactate secretion in melanoma cells (Gottfried et al., 2013). One possible explanation is that diclofenac directly targets the lactate transporter MCT4 which has been suggested by Sasaki et al. in colon carcinoma cells (Sasaki et al., 2016). Furthermore, an inhibitory effect of diclofenac on the uptake of lactic acid has been demonstrated by Emoto et al. (2002).

The glycolytic inhibitor 2-DG has already been tested in clinical trials with limited efficacy. Raez et al. (2013) investigated the maximum tolerated dose of 2-DG in combination with docetaxel and achieved median 2-DG plasma concentrations of 116 ug/ml (about 0.7 mM) in patients with advanced solid tumors. As most *in vitro* studies use concentrations above 1 mM 2-DG, this indicates that effective 2-DG concentrations will most likely not be reached in patients. Therefore novel compounds that can target glycolysis in patients are warranted. In our experiments 0.1 mM diclofenac was even more effective than 10 mM 2-DG in decreasing lactate secretion, a concentration which is achievable in patients (Drugs.com, 2018). Based on these data, we conclude that the described inhibitory effects of diclofenac on tumor cells *in vitro* and in murine tumor models may in part relate not only to COX inhibition but also to glycolysis inhibition (Pantziarka et al., 2016).

Diflunisal is a difluorophenyl derivative of salicylic acid which has been shown to be an effective and well-tolerated analgesic for long-term use. In contrast to diclofenac it has a long half-life and prolonged intake of 500 mg b.i.d. for 11 days results in mean peak plasma levels of 190 ± 33 μg/mL (about 0.8 mM, Drugs.com, 2018). In our hands high concentrations of 0.8 mM diflunisal alone could inhibit proliferation of AML cell lines and primary blasts but induction of apoptosis was only achieved in combination with low dose diclofenac and metformin. Recently it has been shown that diflunisal can inhibit the growth of AML1-ETO positive leukemia cells in SCID mice via specific inhibition of CBP/p300 acetyl-transferase activity. Competition of diflunisal with acetyl-CoA at the catalytic side blocks acetylation of histones and non-histone proteins (Shirakawa et al., 2016). CBP/p300 also acetylates transcriptional regulators such as c-MYC and acetylation decreases ubiquitination, which results in stabilization of c-MYC proteins (Vervoorts et al., 2003). These findings suggest a possible link between c-MYC destabilization by diflunisal and its impact on tumor proliferation as c-MYC directly regulates glucose metabolic enzymes as well as genes involved in mitochondrial biogenesis (Gao et al., 2009). Beside these non-classical effects of diflunisal, it could probably also reduce AML proliferation via COX-2 inhibition as several reports show that COX-2 inhibition limits leukemia growth (Zhang et al., 2013; Hua et al., 2014).

Metformin is commonly used for the treatment of type 2 diabetes and epidemiological studies indicate that tumor incidence is lower in diabetic patients taking metformin (Heckman-Stoddard et al., 2017). In the acute pro-myelocytic leukemia (APL) cell line NB4 metformin has been shown to induce degradation of c-Myc and to induce apoptosis, however, only at concentrations of 5 mM and higher (Huai et al., 2012). In another APL cell line HL-60 low metformin concentrations of 0.4 mM in combination with paclitaxel could induce apoptosis (Asik et al., 2018). In our experiments metformin alone had neither an effect on tumor cell proliferation nor on apoptosis even at concentrations up to 10 mM but OXPHOS activity was clearly suppressed in AML cell lines and primary blasts. This indicates that metformin reprograms tumor cell metabolism and shifts energy production to glycolysis. In line, lactate secretion was enhanced by metformin treatment. This may explain the synergistic anti-proliferative effect of diclofenac and metformin as diclofenac lowers the glycolytic activity.

Targeting OXPHOS has also been suggested by Farge et al. (2017) for chemotherapy-resistant AML cells. Here, cytarabine-resistant AML cells exhibited a specific metabolic phenotype characterized by high OXPHOS. Targeting OXPHOS with 10 mM metformin enhanced the antileukemic effects of 2 μM cytarabine. We combined low dose 0.02 μM cytarabine, the most effective cytostatic drug used in the treatment of AML patients, with metformin and NSAIDs. Cytarabine as a single drug exhibited clear effects on proliferation of leukemia cell lines and primary AML blasts, however, the impact on apoptosis induction was limited. Combination with NSAIDs clearly accelerated apoptosis induction suggesting that combination of low dose chemotherapy with metabolic targeting is an effective strategy.

In summary, there is increasing evidence that not only solid tumors but also leukemia, exhibit changes in metabolism which could be used for metabolic targeting. Beside AMLs with increased glycolytic activity (Ju et al., 2017) other reports state that OXPHOS is of specific importance for AML cells (Farge et al., 2017). These data suggest a broad heterogeneity in oxidative and metabolic requirements in AML cells. Therefore, induction of anakoinosis with combined metabolic targeting of glycolysis, COX and mitochondrial activity together with low dose standard chemotherapy might present a promising therapeutic strategy in AML.

## Conclusion

This study has shown that anti-metabolic treatment can be used to inhibit AML cell line and primary blast proliferation and induce apoptosis in physiologically achievable concentrations. These findings suggest that targeting tumor cell metabolism is an option not only for solid tumors but also hematological malignancies.

## Author Contributions

KR and MK designed the experiments and wrote the manuscript. KS and KP analyzed the data and wrote the manuscript. AnS, NK, IU, PS, S-MD, AsS, KH, MF, CB, and CBrum were involved in experiments and data collection. SK, ST, and EG designed the protocols and discussed data.

## Conflict of Interest Statement

The authors declare that the research was conducted in the absence of any commercial or financial relationships that could be construed as a potential conflict of interest.
